# The IDO1 selective inhibitor epacadostat enhances dendritic cell immunogenicity and lytic ability of tumor antigen-specific T cells

**DOI:** 10.18632/oncotarget.9326

**Published:** 2016-05-12

**Authors:** Caroline Jochems, Massimo Fantini, Romaine I. Fernando, Anna R. Kwilas, Renee N. Donahue, Lauren M. Lepone, Italia Grenga, Young-Seung Kim, Martin W. Brechbiel, James L. Gulley, Ravi A. Madan, Christopher R. Heery, James W. Hodge, Robert Newton, Jeffrey Schlom, Kwong Y. Tsang

**Affiliations:** ^1^ Laboratory of Tumor Immunology and Biology, Center for Cancer Research, National Cancer Institute, National Institutes of Health, Bethesda, MD, USA; ^2^ Radioimmune Inorganic Chemistry Section, Radiation Oncology Branch, Center for Cancer Research, National Cancer Institute, National Institutes of Health, Bethesda, MD, USA; ^3^ Genitourinary Malignancies Branch, Center for Cancer Research, National Cancer Institute, National Institutes of Health, Bethesda, MD, USA; ^4^ Incyte Corporation, Wilmington, DE, USA

**Keywords:** IDO inhibitor, dendritic cells, T cells, indoleamine-2, 3-dioxygenase (IDO), Tregs

## Abstract

Epacadostat is a novel inhibitor of indoleamine-2,3-dioxygenase-1 (IDO1) that suppresses systemic tryptophan catabolism and is currently being evaluated in ongoing clinical trials. We investigated the effects of epacadostat on (a) human dendritic cells (DCs) with respect to maturation and ability to activate human tumor antigen-specific cytotoxic T-cell (CTL) lines, and subsequent T-cell lysis of tumor cells, (b) human regulatory T cells (Tregs), and (c) human peripheral blood mononuclear cells (PBMCs) *in vitro*. Simultaneous treatment with epacadostat and IFN-γ plus lipopolysaccharide (LPS) did not change the phenotype of matured human DCs, and as expected decreased the tryptophan breakdown and kynurenine production. Peptide-specific T-cell lines stimulated with DCs pulsed with peptide produced significantly more IFN-γ, TNFα, GM-CSF and IL-8 if the DCs were treated with epacadostat. These T cells also displayed higher levels of tumor cell lysis on a per cell basis. Epacadostat also significantly decreased Treg proliferation induced by IDO production from IFN-γ plus LPS matured human DCs, although the Treg phenotype did not change. Multicolor flow cytometry was performed on human PBMCs treated with epacadostat; analysis of 123 discrete immune cell subsets revealed no changes in major immune cell types, an increase in activated CD83^+^ conventional DCs, and a decrease in immature activated Tim3^+^ NK cells. These studies show for the first time several effects of epacadostat on human DCs, and subsequent effects on CTL and Tregs, and provide a rationale as to how epacadostat could potentially increase the efficacy of immunotherapeutics, including cancer vaccines.

## INTRODUCTION

Indoleamine-2,3-dioxygenase-1 (IDO1) is an intracellular immunoregulatory enzyme that contributes to immunosuppression, tolerance and tumor escape by catabolizing tryptophan. It is the first and rate-limiting step in tryptophan (Trp) degradation, leading to subsequent production of kynurenines (Kyn). Studies in human *in vitro* systems have shown that depletion of tryptophan leads to an immunosuppressive tumor environment through (a) amino acid starvation of T cells, which leads to inhibition of T-cell proliferation [1–3], and (b) accumulation of Trp metabolites such as Kyn and kynurenic acid, which bind to the cytoplasmic transcription factor aryl hydrocarbon receptor (AhR) [4], leading to differentiation of naïve CD4^+^ T cells into regulatory T cells (Tregs) and suppression of T_H_17 cells [5–7], as well as promotion of a tolerogenic dendritic cell (DC) phenotype through action on IDO^NEG^ DCs [3]. AhR also induces IDO-production by human DCs in a feedback loop that further inhibits T-cell proliferation [3]. The role of AhR on CD8^+^ T cells is not yet known. The role of AhR in controlling disease tolerance and generation of Tregs has also been studied in mice [4, 8]. Expression of functional IDO enzyme has been demonstrated in multiple human tumors of various origin [9], in DCs [10], macrophages [2], and in plasmacytoid DCs in tumor-draining lymph nodes [11]. IDO-expression has been associated with decreased immune cell infiltration and an increased infiltration of Tregs in tumors [12]. A high expression of IDO has been associated with increased frequencies of metastasis in patients with colorectal carcinoma [13], hepatocellular carcinoma [14], and endometrial tumors [15], and with invasive uterine cervical cancer [16]. IDO-expression also increases as melanoma progresses [17] and has been identified as an independent prognostic marker of survival in several cancers. Low IDO-expression correlated with longer overall survival in patients with hepatocellular carcinoma [14], endometrial cancer [15], and non-small-cell lung cancer [18]. In addition, IDO has been identified as a critical resistance mechanism in anti-tumor immunotherapy targeting the immune checkpoint CTLA-4 [19].

Inhibition of IDO is a very promising area of cancer immunotherapy, and three drugs that are currently in clinical trials are 1-methyl-tryptophan (1-MT), NLG919, and epacadostat. 1-MT was first described as an IDO inhibitor in 1991 [20], and is now being tested in clinical trials as 1-methyl-D-tryptophan (indoximod and NLG8189). Oral indoximod has been well tolerated alone or in combination with docetaxel, and there have been some objective responses [21, 22]. Epacadostat is an orally active hydroxyamidine small molecule inhibitor, which selectively inhibits the enzymatic activity of IDO1, with little or no activity against IDO2 and TDO (tryptophan-2,3-dioxygenase) [23, 24]. It competitively blocks Trp binding to IDO1 and its subsequent degradation to Kyn, thus increasing Trp levels and decreasing the accumulation of metabolites. *Ex vivo* lipopolysaccharide (LPS) plus IFN-γ stimulation of whole blood samples from patients enrolled on a phase I trial in advanced cancers recently showed that > 90% inhibition of IDO1 could be achieved in a dose-dependent manner, and it was well tolerated with grade 1-2 fatigue as the most common adverse event [25, 26].

In the studies reported here the use of IFN-γ in combination with LPS for IDO induction in DCs was used to maximize the IDO activity from DCs to investigate the effects of the epacadostat inhibitor. The studies reported here were conducted *in vitro* to investigate the effects of epacadostat on (a) human DCs with respect to maturation and antigen presentation as determined by phenotypic analysis, (b) activation of tumor antigen-specific cytotoxic T cells (CTL), and their subsequent lysis of tumor cells, (c) Treg proliferation and function, and (d) treatment of human peripheral blood mononuclear cells (PBMCs) and analysis of 123 discrete immune cell subsets.

## RESULTS

### Maturation of human DCs with IFN-γ plus LPS resulted in the highest levels of IDO1 mRNA and IDO intracellular expression

**Figure 1:**
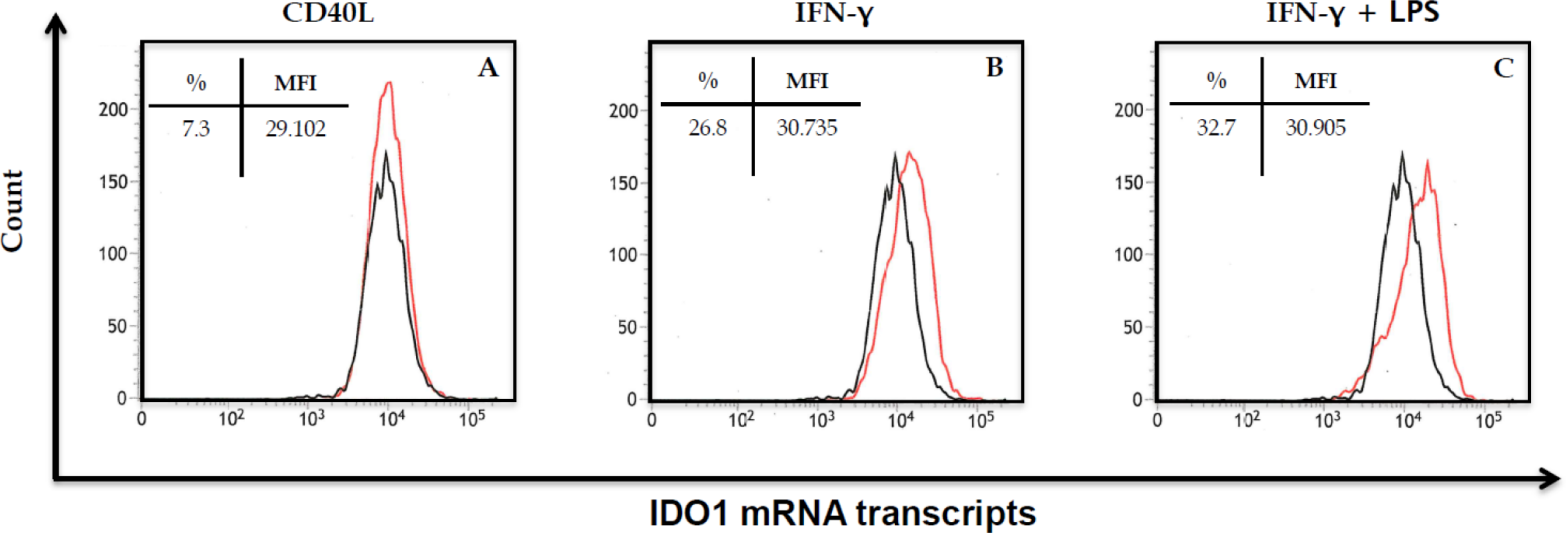
Detection of IDO1 mRNA transcripts in mature dendritic cells Dendritic cells were generated from a healthy donor, and matured with CD40L (1 μg/ml) for 24 hours (**A**), IFN-γ (50 ng/ml) for 48 hours (**B**), or IFN-γ (50 ng/ml) plus LPS (1 μg/ml) for 48 hours **(C)**. IDO1 mRNA transcripts were detected using the human PrimeFlow^™^ RNA assay. Results are expressed as percent IDO1 mRNA^+^ cells in the CD11c^+^ population (red) compared to immature dendritic cells (black: 6.9%). The experiment was repeated in 3 additional donors with similar results.

Human DCs for all experiments were generated from healthy donors as described in Materials and Methods, and used for subsequent experiments after maturation. We first wanted to evaluate the most effective way to mature the DCs to induce maximum production of IDO1. DCs were subjected to flow cytometry either immature or after maturation with CD40L (24 hours), IFN-γ (50 ng/ml) or IFN-γ (50 ng/ml) plus LPS (1 μg/ml) (48 hours). As seen in Table 1, maturation with IFN-γ or IFN-γ plus LPS increased the expression of IDO1 by intracellular staining compared to both immature cells and cells matured with CD40L. Maturation with IFN-γ plus LPS also resulted in the highest levels of the DC activation markers CD80 and CD83. Thus for all further studies, DCs were matured with the combination of IFN-γ and LPS to induce maximal IDO1-production. To confirm the increased expression of IDO1 in IFN-γ plus LPS matured DCs, the human PrimeFlow™ RNA Assay was used to detect IDO1 mRNA transcripts. As can be seen in Figure 1, maturation with CD40L, IFN-γ, or IFN-γ plus LPS resulted in IDO1 mRNA transcripts in 7.3%, 26.8% and 32.7% of DCs, respectively.

**Table 1:** Maturation of human dendritic cells with IFN-γ plus LPS resulted in higher levels of intracellular IDO1 expression

Dendritic cells	IDO1^+^ (%)	CD80^+^ (%)	CD83^+^ (%)
Immature	8.0	19.9	37.0
CD40L	8.3	48.3	73.9
IFN-γ	26.8	52.6	71.1
IFN-γ plus LPS	29.4	77.6	80.5

Human DCs were generated from a healthy donor. Immature DCs and DCs matured with CD40L (24 hours), IFN-γ (48 hours), or IFN-γ plus LPS (48 hours) were analyzed for the expression of IDO1, CD80 and CD83 by flow cytometry. Results are expressed as % positive cells of all CD11c^+^ DCs in 1 out of 3 donors assayed.

Studies were undertaken to determine if epacadostat changed the expression levels of activation markers on matured human DCs. DCs were prepared from two different healthy donors. After 5 days, the DCs were matured by the addition of IFN-γ plus LPS for 48 hours to induce IDO1 expression, and various concentrations of epacadostat were added simultaneously. Our initial studies showed that epacadostat could more efficiently inhibit IDO1-production when added concurrently with IFN-γ and LPS, and that incubation for 48 hours was preferable to 24 hours. After 48 hours, cells were detached and subjected to phenotypic analysis by flow cytometry. There were no significant changes in the expression levels of CD80, CD83, programmed cell death protein-1 (PD-1), programmed cell death protein-1 ligand (PD-L1), or B7-DC (PD-L2) between cells treated with various concentrations of epacadostat (Table 2A). The presence of active IDO1 enzyme can be measured by evaluation of the levels of Trp and the levels of its metabolite Kyn, as well as the Kyn/Trp ratio, by HPLC. Trp and Kyn were measured in the supernatants of immature DCs and DCs matured with IFN-**γ** or IFN-γ plus LPS, treated with or without epacadostat. As seen in Table 2B, the Trp level (expressed as the percent of the original amount of Trp that remains) was 75.8% in supernatants of immature DCs with little breakdown to Kyn (24.2%) and a Kyn/Trp ratio of 0.32. In contrast, in supernatants of matured DCs without the inhibitor, there was appreciable breakdown of Trp, with a Kyn/Trp ratio of 5.9 for the DCs matured with IFN-γ, and 9.2 for DCs matured with IFN-γ plus LPS. Treatment with 1 μM of epacadostat resulted in almost no breakdown of Trp, and Kyn levels as low as 3.3% and 0%, respectively.

**Table 2A:** Treatment with epacadostat did not change the expression levels of CD80, CD83, PDL1, PD-1, or B7-DC (PD-L2) on IFN-γ plus LPS matured human DCs

HD	Epacadostat (μM)	CD80^+^	CD80^+^ CD83^+^	CD80^+^ PD-L1^+^	CD80^+^ PD-1^+^	CD80^+^ B7-DC^+^
# 1	0	90.3	64.0	90.3	0.09	34.9
# 1	0.05	91.6	67.3	91.6	0	33.5
# 1	0.25	93.9	72.2	93.9	0.09	30.1
# 1	1.0	92.6	69.8	92.6	0	30.0
# 2	0	92.5	71.8	92.5	0	32.0
# 2	0.05	94.7	77.8	94.7	0	31.3
# 2	0.25	95.0	78.7	95.0	0	31.0
# 2	1.0	94.5	77.0	94.5	0	31.9

**Table 2B:** Treatment with epacadostat decreased the breakdown of tryptophan by matured human DCs

DCs	IFN-γ plus LPS	Epacadostat (μM)	Kyn	Trp	Kyn / Trp ratio
Immature	**-**	**-**	24.2	75.8	0.32
Mature	IFN-γ	0	85.5	14.8	5.9
Mature	IFN-γ	1.0 μM	3.3	96.7	0.03
Mature	IFN-γ plus LPS	0	90.2	9.8	9.2
Mature	IFN-γ plus LPS	1.0 μM	0	100	-

**A**. Human DCs were generated from 2 healthy donors (HD #1 and #2). PBMCs were plated for 2 hours at 2 × 10^7^ PBMCs/well in a 6-well plate in AIM-V medium. After careful washing, the adherent monocytes were cultured for 5 days in AIM-V medium containing rhGM-CSF (100 ng/ml) and rhIL-4 (20 ng/ml). Immature DCs were then treated for 48 hours with IFN-γ (50 ng/ml) and LPS (1 μg/ml) with or without epacadostat at different concentrations. After trypsinization and careful scraping, DCs were subjected to flow cytometry. Results are expressed as % positive cells of all DCs. **B**. The concentrations of tryptophan (Trp) and kynurenine (Kyn) were evaluated by HPLC in 48 hours in supernatants of immature DCs or DCs matured with IFN-γ or IFN-γ plus LPS, and treated with or without epacadostat for 48 hours. Results are expressed as the % of the original amount of Trp that has been catabolized (Kyn) or that remains (Trp), and as the Kyn/Trp ratio.

### Effect on antigen-specific T cells after stimulation with peptide-pulsed DCs treated with epacadostat

A CEA-specific T-cell line derived from a cancer patient was stimulated as previously described [27] using CEA-peptide pulsed DCs exposed to 0, 0.25 or 1.0 μM of epacadostat. As seen in Table 3A, T-cell stimulation with peptide-pulsed DCs treated with epacadostat resulted in significantly higher levels of IFN-γ in supernatants, as compared to those not treated with the IDO-inhibitor. Additional cytokines were measured in a similar experiment using a C-terminus of mucin-1 (MUC1-C)–specific T-cell line; after treating the DCs with 0 or 1.0 μM of epacadostat before peptide-pulsing them, levels of IFN-γ, GM-CSF, IL-8, and TNFα were found to have increased (Table 3B). These data thus demonstrated that treating DCs with the IDO-inhibitor epacadostat increased their capacity to stimulate CD8^+^ antigen-specific T-cell lines *in vitro*.

**Table 3A and 3B:** Peptide-specific T cells produced increased levels of IFN-γ after stimulation with peptide-pulsed DCs exposed to epacadostat

A				
	CEA peptide	Epacadostat		
		0 μM	0.25 μM	1.0 μM
DCs + T cells	−	< 15.6	< 15.6	< 15.6
DCs + T cells	+	**291**	**602**	**2,750**
DCs	−	< 15.6	NA	< 15.6
DCs	+	< 15.6	NA	< 15.6

B					
	Epacadostat	IFN-γ	GM-CSF	IL-8	TNFα
DCs + T cells	0	42	124	29	7.5
DCs + T cells	1.0 μM	669	1,745	412	191

A MUC1-C–specific HLA-A24^+^ T-cell line derived from a patient with prostate cancer was stimulated using its specific MUC1 peptide and DCs treated with 0, 0.25 or 1.0 μM of epacadostat. Five days after stimulation, the T cells were used in a CTL assay using PC3 (human prostate carcinoma, MUC1^+^, HLA-A24^+^) as a target and ASPC-1 (human pancreatic carcinoma, MUC1^+^, HLA-A24^NEG^) as a negative control. Pre-treating the DCs with epacadostat resulted in increased tumor cell lysis (Table 3C).

**Table 3C:** Lysis of human tumor cells increased after peptide-specific T-cell lines were stimulated with peptide-pulsed DCs exposed to epacadostat

T-cell line	DC:T cell ratio	Epacadostat μM	PC3 % lysis (SD)	MDA-MB231 % lysis (SD)	ASPC-1 % lysis (SD)
**MUC1-C**	**1:5**	0	41.0 (4.5)	-	0
		0.25	58.1 (4.3)^*^	-	2.0 (1.1)
		1.0	56.7 (7.5)^*^	-	1.8 (0.1)
**MUC1-C**	**1:1**	0	54.8 (4.4)	-	1.3 (0.1)
		0.25	69.6 (3.4)^*^	-	2.4 (0.3)
		1.0	64.6 (3.9)^*^	-	1.5 (0.5)
**Brachyury**	**1:5**	0	-	3.5 (0.6)	0.2 (0.2)
		1.0	-	26.9 (1.6)^*^	6.1 (1.6)

**A**. A CEA-specific T-cell line was stimulated using CEA peptide and DCs exposed to 0, 0.25 or 1.0 μM of epacadostat as previously described [27]. Stimulation with DCs exposed to epacadostat resulted in significantly higher levels of IFN-γ in supernatants after 24 hours. NA: not available. **B**. A MUC1-C-specific T-cell line produced significantly higher levels of cytokines after 24 hours stimulation with peptide-pulsed DCs exposed to epacadostat. **C**. A MUC1-C–specific, HLA-A24^+^ T-cell line derived from a patient with prostate cancer was stimulated using its specific MUC1-C peptide and DCs exposed to 0, 0.25 or 1.0 μM of epacadostat. Five days after stimulation, the T cells were used in a CTL assay using PC3 (human prostate carcinoma, MUC1^+^, HLA-A24^+^) as a target and ASPC-1 (human pancreatic carcinoma, MUC1^+^, HLA-A24^NEG^) as a negative control. Results are expressed as % lysis (SD) at an effector:target ratio of 30:1. A brachyury-specific, HLA-A2^+^ T-cell line derived from another patient with prostate cancer was stimulated using its specific peptide and DCs exposed to 0, or 1.0 μM of epacadostat and used in a CTL assay using MDA-MB231 (human breast carcinoma, brachyury^+^, HLA-A2^+^) as a target and ASPC-1 as a negative control. Results are expressed as % lysis (SD) at an effector:target ratio of 60:1.

* *P* < 0.05 by paired *t*-test compared to no treatment. The experiments have been repeated multiple times, and the table shows one of them.

An additional HLA-A2–restricted T-cell line, specific for brachyury peptide, was derived from another prostate cancer patient; after stimulation with epacadostat (1 μM)– treated DCs, the lysis of the human breast cancer cell line MDA-MB231 increased from 3.5% to 26.9% (Table 3C). These results thus demonstrated for the first time that epacadostat treatment of DCs prior to peptide-pulsing resulted in both increased cytokine production and increased tumor cell lysis by antigen-specific CD8^+^ T-cell lines derived from cancer patients.

### Effect of epacadostat on PBMC immune cell subsets

Human PBMCs from 10 healthy donors were analyzed by multiparameter flow cytometry after 48 hours incubation with epacadostat (0, 0.25 or 1.0 μM). A total of 123 peripheral immune cell subsets were examined for changes following treatment (see Supplementary Table 1). These included nine standard parental immune cell types (CD4^+^ and CD8^+^ T cells, Tregs, B cells, natural killer (NK) cells, NKT cells, conventional dendritic cells (cDCs), plasmacytoid DCs (pDCs), and myeloid derived suppressor cells (MDSCs)), and 114 subsets of these cell types relating to maturation and function. All of the nine standard immune cell types were unchanged following epacadostat treatment (Table 4). Of the 114 subsets relating to maturation and function, only two were changed following treatment with 1 μM of epacadostat. There was a significant increase (from 0.009 to 0.011% of PBMCs, *P* = 0.02) in cDCs expressing CD83, a marker of activation, with seven out of 10 donors having a greater than 25% increase. There was also a significant decrease (from 0.007 to 0.004% of PBMCs, *P* = 0.02) in immature NK cells expressing T-cell immunoglobulin and mucin domain-3 (Tim-3)^+^, a marker of activation, with seven out of 10 donors having a greater than 25% decrease. However, as the frequencies of these subsets are low as a percent of total PBMCs, these changes are considered to be only notable trends. Thus, treatment of PBMCs with epacadostat resulted in little or no change in the phenotype of any of 123 immune cell subsets in the absence of any additional stimulation.

**Table 4:** Standard immune cell types in healthy donors following 48 hours treatment with epacadostat

Immune cell type	Epacadostat 0	Epacadostat 0.25 μM	Epacadostat 1 μM	*P*-value
CD4^+^	38	38	38	0.85
CD8^+^	27	27	26	0.56
Tregs	1.2	1.2	0.9	0.28
B cells	10	10	11	0.69
NK	1.7	1.6	1.6	0.08
NKT	0.7	0.7	0.7	0.43
cDC	0.16	0.17	0.16	0.37
pDC	0.004	0.005	0.004	0.77
MDSC	0.6	0.5	0.5	0.32

Human PBMCs from 10 healthy donors were subjected to multiparameter flow cytometric evaluation after 48 hours incubation with epacadostat (0, 0.25 or 1.0 μM). Nine standard immune cell types were analyzed. Values are displayed as the median frequency of PBMCs. There were no significant changes between the treatment groups (Wilcoxon matched-pairs signed rank test).

cDC, conventional dendritic cells; MDSC, myeloid derived suppressor cells; NK, natural killer; NKT, natural killer T cells; PBMCs, peripheral blood mononuclear cells; pDC, plasmacytoid DC; Tregs, regulatory T cells.

### Effect of epacadostat on Tregs

Dendritic cells were generated from two healthy donors, matured with IFN-γ plus LPS with or without epacadostat, and then used for co-culture with autologous isolated CD4^+^ T cells for 5 days, after which flow cytometry was performed. As seen in Table 5A, there were no significant differences in the expression levels of FoxP3, CTLA-4 or CCR4 on Tregs (defined as CD4^+^, CD25^+^, CD127^NEG^, FoxP3^+^) after 5 days in culture with DCs exposed to epacadostat (1.0 μM) or DCs treated only with IFN-γ plus LPS (Table 5A).

**Table 5A:** Phenotypic analysis of regulatory T cells from healthy donors after co-culture with autologous DCs matured with IFN-γ plus LPS and treated with epacadostat

Donor	Epacadostat μM	% CTLA-4^+^ / Tregs	FoxP3 MFI	% CCR4^+^ / CD4	CCR4 MFI
**#1**	0	7.7	1,576	9.3	11,126
	1.0	9.2	1,553	8.1	10,498
**#2**	0	3.6	1,449	12.5	8,404
	1.0	3.3	1,349	11.8	7,945

A proliferation assay of similarly treated Tregs or CD4^+^ T cells with antigen-presenting cells (APCs) from freshly thawed PBMCs from the same donor was performed. As can be seen in Table 5B, maturation of DCs with IFN-γ plus LPS without the IDO-inhibitor resulted in four times more Tregs per plate compared to maturation with CD40L. The number of Tregs decreased when epacadostat was added. The proliferation rate as measured by counts per minute (CPM) also increased for Tregs after maturation with IFN-γ plus LPS, and decreased upon treatment with epacadostat. Taken together, these results demonstrated that epacadostat decreased Treg proliferation without altering the Treg phenotype.

**Table 5B:** IDO-producing human DCs induce proliferation of Tregs, which can be inhibited by epacadostat

Maturation	Epacadostat μM	Tregs Cell count	Tregs CPM	CD4 T cells CPM	APCs CPM
CD40L	0	1.2 × 10^5^	5,564	15,566	1,317
IFN-γ plus LPS	0	4.9 × 10^5^	46,290	23,411	778
IFN-γ plus LPS	0.25	3.3 × 10^5^	21,308	15,353	675
IFN-γ plus LPS	1.0	2.6 × 10^5^	22,631	23,385	834

**A**. DCs were generated from 2 healthy donors, matured with IFN-γ plus LPS with or without epacadostat, and then used for co-culture with autologous purified CD4^+^ T cells for 5 days. Flow cytometry was performed after 5 days. Tregs were defined as CD4^+^, CD25^+^, CD127^NEG^, and FoxP3^+^ cells. Results are shown as frequency and MFI. **B**. Dendritic cells were generated from a healthy donor and matured with IFN-γ plus LPS to induce IDO production as described in Materials and Methods. CD4^+^ T cells were isolated from the same donor and added to the culture at 2 × 10^6^/well in 6 wells each with 0, 0.25 or 1.0 μM of epacadostat. Five days later, Tregs were isolated from the cultures and counted, and a proliferation assay using these Tregs with autologous purified CD4^+^ T cells and irradiated APCs from freshly thawed PBMCs was performed. Results are shown as the cell count for Tregs (isolated CD4^+^, CD25^+^, CD127^NEG^) after the first proliferation of a total of 12 × 10^6^ CD4^+^ T cells, as well as the counts per minute from the proliferation of Tregs (10^4^/well), CD4^+^ T cells (CD4^+^, CD25^NEG^) (10^4^/well), and APCs (irradiated PBMCs left over after isolation of T cells) (10^5^/well). Results shown are from 1 healthy donor out of several assayed.

## DISCUSSION

The IDO1 enzyme inhibitor epacadostat represents a new class of inhibitor, a hydroxyamidine small molecule, which potently inhibits IDO1 function without affecting IDO2 and TDO. It has been very well tolerated in clinical trials. It was evaluated in a phase I dose escalation study (NCT01195311), and led to stable disease for more than 8 weeks in 30% of the 52 enrolled patients with refractory disease. The trial did not reach dose-limiting toxicity, and the recommended monotherapy dose is 600 mg twice daily. Preliminary results from a phase I/II combination therapy trial with epacadostat and ipilimumab in melanoma patients showed 6/8 patients with tumor reduction by the first evaluation [28]. There are currently 13 ongoing trials with epacadostat alone or in combination therapy in ovarian, fallopian tube, and primary peritoneal cancer, melanoma, non-small cell lung cancer, myelodysplastic syndromes and advanced solid tumors. Preclinically, combination therapy with epacadostat and anti-CTLA-4 or anti-PD-1/PD-L1 improved tumor control and increased IL-2 production and CD8 T-cell proliferation in murine melanoma better than single agent therapy [29]. The finding of cytotoxic T cells specific for IDO1 in both healthy individuals and cancer patients provides further evidence for the immunogenicity of IDO1 [30]. These cells are capable of lysing tumor cells and DCs expressing IDO1, which boosts the immune response [30, 31]. A clinical trial evaluating a vaccine containing a peptide derived from IDO was recently completed in patients with metastatic malignant melanoma (NCT02077114). Common approaches for evaluation of IDO activity include (a) IDO1 protein expression by Western blot, immunohistochemistry and/or flow cytometry, (b) IDO1 mRNA expression, and (c) determination of Trp consumption and Kyn accumulation; this determination of Trp consumption and Kyn accumulation by HPLC is indispensible. We have analyzed IDO activity employing all three approaches to investigate the ability of the IDO1 inhibitor epacadostat to reverse the functional activity of the IDO1 enzyme.

Epacadostat has been shown to increase the survival of human DCs and decrease DC apoptosis, and to enhance CD4^+^ and CD8^+^ T-cell proliferation and IFNγ production, which could potentially result in more efficient priming of T cells [24]. We therefore wanted to evaluate its effects on DC stimulation of tumor associated antigen-specific cytotoxic T-cell lines *in vitro*. As expected, epacadostat treatment of DCs decreased the breakdown of Trp and accumulation of Kyn in supernatants. We found no phenotypic changes in the epacadostat-treated matured DCs by flow cytometry. We show for the first time that pre-treating human DCs with epacadostat before they were used to stimulate peptide-specific T-cell lines derived from cancer patients resulted in increased production of IFN-γ and increased lysis of human tumor cell line targets expressing various tumor-associated antigens.

It has previously been shown in the P815 murine tumor model that potentially immunogenic tumor cells that express IDO1 are protected from immune-mediated rejection, and that treatment with the IDO-inhibitor 1-MT can revert this effect [9]. Ito et al. previously found that the induction of HBsAg-specific CTL in mice after vaccination was upregulated by IDO inhibition using 1-MT [32]. The increase in tumor cell lysis observed in the study reported here after CTL stimulation with epacadostat-treated DCs, and the potentially increased immunogenicity of tumor cells observed after IDO-inhibitor treatment, thus provide a rationale for combination therapy of epacadostat with cancer vaccines able to induce T-cell anti-tumor activity. It has also been demonstrated that IDO1 expression and activity can lead to the generation of reactive oxygen species (ROS) in various cell types in both humans and mice [33, 34]. Treatment of NK cells with Kyn can induce growth inhibition and apoptosis of human NK cells, and inhibit anti-tumor activity of NK cells against IDO positive cancer cells. Kyn-induced NK-cell apoptosis in humans is primarily through an ROS-mediated pathway [35, 36]. In addition, it has been suggested that ROS can inhibit anti-cancer activity of T cells and lead to cell death of cytotoxic T cells [37, 38]. The IDO1 inhibitor epacadostat can interact with NK cells as well as CTLs and IDO1-producing tumor cells in regulating the immune responses.

IDO1-expression is associated with increased differentiation of naïve CD4 T cells into Tregs in humans [5–7], and with increased infiltration of Tregs in tumor [12]. We show here that Treg proliferation was increased in the presence of IDO1, and treatment with epacadostat decreased the Treg proliferation. There were no phenotypic changes to Tregs after treatment. It has further been suggested that one mechanism by which IDO can enhance Treg activity is by inhibiting Tregs from undergoing phenotypic reprogramming. Reprogramming in the CD4^+^ lineage refers to the alteration of the mature and differentiated CD4^+^ subsets to acquire phenotypic and functional characteristics of other subsets [39]. IDO1 can block Treg reprogramming at least in part by inhibition of IL-6 production [40]. IL-6 is an important cytokine driving the abrogation of functional Treg suppressive activity *in vivo* [41], and reprogramming of Tregs into IL-17–producing T_H_17 cells *in vitro* [42].

In a recent review of IDO pathway inhibitors [43], the authors conclude that IDO-inhibition has been well tolerated in cancer patients, with clinical anticancer effects seen in a subset of patients, and that combination therapy with chemotherapy, radiotherapy or immunotherapy may be effective against a wide range of malignancies. The immune enhancing data reported here support the combination of epacadostat with such therapies. The combined decrease in Treg proliferation and increase in CTL activity seen with IDO1 inhibition supplies the rationale for clinical studies employing the combination of epacadostat and cancer vaccines or other immunotherapeutics. We also demonstrate for the first time that *in vitro* treatment of human PBMCs with epacadostat did not change any peripheral immune cell subsets in a way that could have a negative biological impact on concurrent vaccine therapy. In future studies, it would be interesting to investigate different cancer types and stages for the ability of epacadostat to enhance dendritic cell immunogenicity and the lytic ability of antigen-specific T cells.

## MATERIALS AND METHODS

### Generation of dendritic cells from PBMCs

Peripheral blood was collected from healthy volunteer donors from the National Institutes of Health Clinical Center Blood Bank (Bethesda, MD, USA) (NCT00001846), and PBMCs were isolated by centrifugation on a density gradient (Lymphocyte Separation Medium, ICN Biochemicals, Aurora, VA). DCs were generated using a modification of the previously described procedure [44]. DCs were cultured in AIM-V medium containing 100 ng/ml GM-CSF and 20 ng/ml IL-4 (PeproTech, Rocky Hill, NJ). After 5 days in culture, the DCs were matured by the addition of 1 μg/ml CD40L and 1μg/ml enhancer (Enzo Life Sciences, Farmingdale, NY) for 24 hours as a control, IFN-γ (50 ng/ml) for 48 hours, or IFN-γ (50 ng/ml) and LPS (1 μg/ml) for 48 hours to induce IDO1 expression. The combination of LPS and IFN-γ led to the highest IDO1 expression, and was chosen for most experiments, since our aim was to evaluate the effects of the IDO-inhibitor epacadostat on the different immune cells, and not to mimic the clinical situation.

### IDO-inhibitor

The IDO1 selective inhibitor epacadostat, obtained through a Cooperative Research and Development Agreement with Incyte Corporation (Wilmington, DE), was dissolved in DMSO and used at different concentrations as described. The concentration used in most experiments, 1.0 μM, was chosen to mimic the serum concentration observed in patients receiving 300 mg BID, where there was > 90% inhibition of IDO1 [25]. When the dose was increased further in patients there was no additional effect, and at lower doses the efficacy was decreased in a dose proportional manner.

### Tumor cell cultures

The human pancreatic carcinoma cell line ASPC-1 (HLA-A1^+^, HLA-A26^+^, MUC1^+^), prostate cancer cell line PC3 (HLA-A24^+^, MUC1^+^), and breast cancer cell line MDA-MB231 (HLA-A2^+^, brachyury^+^) were purchased from American Type Culture Collection (Manassas, VA). All cell cultures were free of mycoplasma and maintained in complete medium (RPMI 1640 supplemented with 10% fetal calf serum, 100 U/ml penicillin, 100 μg/ml streptomycin and 2 mM L-glutamine; Mediatech, Herndon, VA).

### T-cell lines and CTL assays

MUC1-, CEA- and brachyury-specific HLA-A2– and HLA-A24–restricted T-cell lines were established and used for evaluation of IFN-γ production and in indium-111 release CTL assays as previously described [27]. For the experiments using MUC1-C-specific T-cell lines and DCs, we utilized PBMCs from two prostate cancer patients enrolled in a previously described clinical trial of PSA-TRICOM vaccine in combination with ipilimumab [45]. The CEA-specific T-cell line was derived from a patient with metastatic carcinoma treated with a recombinant CEA-vaccinia vaccine in a phase I trial [46]. In all experiments with antigen-specific T-cell lines, the DCs were derived from HLA-matched healthy donors. An Institutional Review Board of the National Institutes of Health (NIH) Clinical Center had approved the procedures, and informed consent was obtained in accordance with the Declaration of Helsinki.

### Detection of cytokines

Autologous DCs matured with IFN-γ plus LPS for 48 hours were pulsed with peptides and incubated with CEA- or MUC1-specific T-cell lines for 24 hours. The supernatants were then analyzed for IFN-γ by ELISA (Invitrogen, Frederick, MD), or by Multi-Array technology (Meso Scale Diagnostics, Gaithersburg, MD) for detection of additional cytokines.

### Flow cytometric analysis

For flow analysis, 5 × 10^5^ cells were stained in 100 μl staining buffer (PBS, 1% BSA) for 30 minutes at 4°C. Events were acquired on an LSRII flow cytometer (BD Bioscience, San Jose, CA) and analysis was performed in FlowJo 9.5.2 (Treestar Inc., Ashland, OR). Antibodies used for flow cytometric analysis were: CD80-FITC (BD), CD83-BV421 (BioLegend, San Diego, CA), HLA-DR-V500 (BD), CD11c-PECy7 (BD), CD11c-BV605 (BioLegend), PD-L1-FITC (clone M1H1, BD), B7-DC (PD-L2)-PerCPEFluor710 (eBioscience, San Diego, CA) and IDO-PE (clone 700838) (R&D Systems, Minneapolis, MN).

### Detection of IDO1 mRNA

The expression of IDO1 mRNA was evaluated by the human PrimeFlow™ RNA assay (eBioscience) per the manufacturer's instructions. Briefly, after surface staining of mature or immature DCs using CD11c-PE-Cy7 (BD) and CD83-BV421 (BioLegend), cells were permeabilized, and then hybridized with the type 4 Human RPL13A Alexa Fluor^®^ 488 (Affymetrix, cat. VA4-13187) and the type 1 IDO 1 Human Alexa Fluor^®^ 647 (Affymetrix, cat. VA1-14451) probes, per the protocol instructions. After hybridization, cells were acquired using the BD FACSVerse™ flow cytometer (BD). Analysis to detect IDO mRNA transcripts was performed using the BD FACSuite™ software (BD).

### High-performance liquid chromatography (HPLC)

All samples were analyzed by reverse-phase HPLC using a Beckman system Gold HPLC (Fullerton, CA) equipped with a 126 solvent module and 168 UV detector (λ = 254 nm) controlled by 32 Karat software and Beckman Ultrasphere column (ODS, 4.6 × 250 mm, 5 μm). The flow rate was 1 ml/min and the mobile phase was isocratic with 90% A (1 mM phosphate buffer, pH 4) and 10% B (methanol) for 20 minutes.

### *In vitro* epacadostat treatment of healthy donor PBMCs

PBMCs from 10 healthy volunteers were obtained from the NIH Clinical Center Blood Bank (NCT00001846), incubated overnight and then treated with 0, 0.25 or 1.0 μM of epacadostat for 48 hours before flow cytometric analysis of 123 distinct immune cell subsets (Supplementary Table 1) using a panel of 30 different markers (Supplementary Table 2) as previously described [47].

## SUPPLEMENTARY MATERIALS TABLES


