# Supplementary material for: The IDO1 selective inhibitor epacadostat enhances dendritic cell immunogenicity and lytic ability of tumor antigen-specific T cells
Source: Oncotarget. 2016 May 12;7(25):37762–72. doi: 10.18632/oncotarget.9326 (PMC5122347; doi:10.18632/oncotarget.9326)
Supplement: Supplementary file 1 [file oncotarget-07-37762-s001.pdf]

The IDO1 selective inhibitor epacadostat enhances dendritic cell immunogenicity and lytic ability of tumor antigen-specific T cells

Supplementary Materials

Supplemental Table S1: Complete list of 123 peripheral immune cell subsets analyzed by flow cytometry

1. Total CD4$^+$ T cells

- PD-L1$^+$ CD4
- PD-1$^+$ CD4
- CTLA-4$^+$ CD4
- Tim-3$^+$ CD4
- 41BB$^+$ CD4
- ICOS$^+$ CD4
    - PD-L1$^+$ ICOS$^+$ CD4
    - PD-1$^+$ ICOS$^+$ CD4
- Total naïve (CCR7$^+$CD45RA$^+$) CD4
    - PD-L1$^+$ naïve CD4
    - PD-1$^+$ naïve CD4
    - CTLA-4$^+$ naïve CD4
    - Tim-3$^+$ naïve CD4
    - 41BB$^+$ naïve CD4
- Total central memory (CCR7$^+$ CD45RA$^-$) CD4
    - PD-L1$^+$ CM CD4
    - PD-1$^+$ CM CD4
    - CTLA-4$^+$ CM CD4
    - Tim-3$^+$ CM CD4
    - 41BB$^+$ CM CD4
- Total effector memory (CCR7$^-$ CD45RA$^-$) CD4
    - PD-L1$^+$ EM CD4
    - PD-1$^+$ EM CD4
    - CTLA-4$^+$ EM CD4
    - Tim-3$^+$ EM CD4
    - 41BB$^+$ EM CD4
- Total EMRA (CCR7$^-$CD45RA$^+$) CD4
    - PD-L1$^+$ EMRA CD4
    - PD-1$^+$ EMRA CD4
    - CTLA-4$^+$ EMRA CD4
    - Tim-3$^+$ EMRA CD4
    - 41BB$^+$ EMRA CD4

2. Total CD8$^+$ T cells

- PD-L1$^+$ CD8
- PD-1$^+$ CD8
- CTLA-4$^+$ CD8
- Tim-3$^+$ CD8
- 41BB$^+$ CD8
    - Total naïve (CCR7$^+$CD45RA$^+$) CD8
    - PD-L1$^+$ naïve CD8
    - PD-1$^+$ naïve CD8
    - CTLA-4$^+$ naïve CD8
    - Tim-3$^+$ naïve CD8
    - 41BB$^+$ naïve CD8
- Total central memory (CCR7$^+$CD45RA$^-$) CD8
    - PD-L1$^+$ CM CD8
    - PD-1$^+$ CM CD8
    - CTLA-4$^+$ CM CD8
    - Tim-3$^+$ CM CD8
    - 41BB$^+$ CM CD8
- Total effector memory (CCR7$^-$ CD45RA$^-$) CD8
    - PD-L1$^+$ EM CD8
    - PD-1$^+$ EM CD8
    - CTLA-4$^+$ EM CD8
    - Tim-3$^+$ EM CD8
    - 41BB$^+$ EM CD8
- Total EMRA (CCR7$^-$CD45RA$^+$) CD8
    - PD-L1$^+$ EMRA CD8
    - PD-1$^+$ EMRA CD8
    - CTLA-4$^+$ EMRA CD8
    - Tim-3$^+$ EMRA CD8
    - 41BB$^+$ EMRA CD8

3. Total Tregs

- PD-L1$^+$ Tregs
- PD-1$^+$ Tregs

- CTLA-4^{+} Tregs
- ICOS^{+} Tregs
- CD45RA^{+} Tregs
- CD49d^{-} Tregs

4. Total B cells

- PD-L1^{+} B cells
- PD-1^{+} B cells

5. Total NK

- PD-L1^{+} NK
- PD-1^{+} NK
- Tim-3^{+} NK
- Total mature (CD16^{+} CD56^{dim}) NK
    - ○ PD-L1^{+} mature NK
    - ○ PD-1^{+} mature NK
    - ○ Tim-3^{+} mature NK
- Total functional intermediate (CD16^{+} CD56^{br}) NK
    - ○ PD-L1^{+} functional intermediate NK
    - ○ PD-1^{+} functional intermediate NK
    - ○ Tim-3^{+} functional intermediate NK
- Total immature (CD16^{-} CD56^{br}) NK
    - ○ PD-L1^{+} immature NK
    - ○ PD-1^{+} immature NK
    - ○ Tim-3^{+} immature NK
- Total unconventional (CD16^{-} CD56^{dim}) NK
    - ○ PD-L1^{+} unconventional NK
    - ○ PD-1^{+} unconventional NK
    - ○ Tim-3^{+} unconventional NK

6. Total NK-T

- PD-L1^{+} NK-T
- PD-1^{+} NK-T
- Tim-3^{+} NK-T

7. Total cDC

- PD-L1^{+} cDC
- PD-1^{+} cDC
- CD83^{+} cDC
- Tim-3^{+} cDC

8. Total pDC

- PD-L1^{+} pDC
- PD-1^{+} pDC
- CD83^{+} pDC
- Tim-3^{+} pDC

9. Total MDSC

- PD-L1^{+} MDSC
- PD-1^{+} MDSC
- CD16^{+} MDSC
- Total monocytic (CD14^{+} CD15^{-}) MDSC
    - ○ PD-L1^{+} mMDSC
    - ○ PD-1^{+} mMDSC
    - ○ CD16^{+} mMDSC
- Total granulocytic (CD14^{-} CD15^{+}) MDSC
    - ○ PD-L1^{+} gMDSC
    - ○ PD-1^{+} gMDSC
    - ○ CD16^{+} gMDSC
- Total lineage negative (CD14^{-} CD15^{-}) MDSC
    - ○ PD-L1^{+} lin neg MDSC
    - ○ PD-1^{+} lin neg MDSC
    - ○ CD16^{+} lin neg MDSC

Nine standard subsets were identified as well as 114 subsets relating to maturation and function within the standard subsets. cDC, conventional dendritic cells; CM, central memory; CTLA-4, cytotoxic T lymphocyte-associated protein-4; EM, effector memory; EMRA, terminally differentiated effector memory; FoxP3, forkhead box P3; gMDSCs, granulocytic mononuclear derived suppressor cells; ICOS, inducible T cell co-stimulator; lin neg MDSCs, lineage negative MDSCs; mMDSCs, monocytic MDSCs; NK, natural killer; pDC, plasmacytoid DC; PD-1, programmed cell death-1; PD-L1, programmed cell death ligand-1; Tim-3, T cell immunoglobulin and mucin domain-3; Tregs, regulatory T cells.

Supplemental Table S2: Antibodies used for 4 panel stain to identify 123 peripheral immune cell subsets

Fluorochrome	Panel 1	Panel 2	Panel 3	Panel 4
**FITC**	CTLA-4 (A3.4H2.H12, LS Bio)	CTLA-4 (A3.4H2.H12, LS Bio)	CD3 (HIT3a, Biolegend)	CD15 (HI98, Biolegend)
**PE**	PD-1 (EH12.2H7, Biolegend)	PD-1 (EH12.2H7, Biolegend)	PD-1 (EH12.2H7, Biolegend)	PD-1 (EH12.2H7, Biolegend)
**PerCP-Cy5.5**	41BB (4B4-1, Biolegend)	ICOS (C398.4A, Biolegend)	CD303 (201A, Biolegend)	CD19 (HIB19, Biolegend)
**PECy7**	PD-L1 (MIH1, BD)	PD-L1 (MIH1, BD)	PD-L1 (MIH1, BD)	PD-L1 (MIH1, BD)
**BV421**	Tim-3 (F38-2E2, Biolegend)	FoxP3 (206D, Biolegend)	Tim-3 (F38-2E2, Biolegend)	CD14 (M5E2, Biolegend)
**V500**	CCR7 (G043H7, Biolegend)	CD49d (9F10, Biolegend)	CD83 (HB15e, BD)	CD16 (3G8, Biolegend)
**BV605**	CD4 (OKT4, Biolegend)	CD4 (OKT4, Biolegend)	CD56 (HCD56, Biolegend)	HLA-DR (L243, Biolegend)
**Dapi**	Live/Dead (Invitrogen)	Live/Dead (Invitrogen)	Live/Dead (Invitrogen)	Live/Dead (Invitrogen)
**APC**	-	CD25 (M-A251, Biolegend)	HLA-DR (L243, Biolegend)	CD33 (WM53, Biolegend)
**AF700**	CD45RA (HI100, Biolegend)	CD45RA (HI100, Biolegend)	CD16 (3G8, Biolegend)	-
**APC Cy7**	CD8 (RPA-T8, Biolegend)	CD127 (eBioRDR5, Ebioscience)	CD1c (L161, Biolegend)	CD11b (M1/70, Biolegend)

Panel 1: $CD4^+$ T cells, $CD8^+$ T cells; Panel 2: Tregs; Panel 3: NK, NK-T, cDC, pDC; Panel 4: B cells, MDSC. Intracellular antibodies are underlined, and clones and companies are listed under each antibody.

cDC, conventional dendritic cells; CTLA-4, cytotoxic T lymphocyte-associated protein-4; FoxP3, forkhead box P3; HLA, human leukocyte antigen; ICOS, inducible T cell co-stimulator; MDSC, myeloid derived suppressor cells; NK, natural killer; pDC, plasmacytoid DC; PD-1, programmed cell death-1; PD-L1, programmed cell death ligand-1; Tim-3, T cell immunoglobulin and mucin domain-3; Tregs, regulatory T cells.
